# Religious leaders as partners in diabetes self-care promotion: opportunities and challenges from Imams' perspectives in Saudi Arabia

**DOI:** 10.3389/fpubh.2026.1830915

**Published:** 2026-06-11

**Authors:** Bandar S. Alharbi, Majed M. Aljabri

**Affiliations:** Community and Psychiatric Mental Health Department, College of Nursing, King Saud University, Riyadh, Saudi Arabia

**Keywords:** diabetes self-care, faith-based health promotion, Imams, religious leaders, type 2 diabetes mellitus

## Abstract

**Background:**

Type 2 diabetes mellitus (T2DM) poses a major public health challenge in Saudi Arabia, where effective management relies heavily on sustained self-care behaviors. Cultural and religious contexts strongly shape health behaviors, and Imams who hold significant moral authority and community trust may represent valuable partners in diabetes self-care promotion. However, little is known about Imams' perspectives on their potential role in this area.

**Methods:**

A qualitative descriptive study was conducted using semi-structured, in-depth interviews with Imams in Saudi Arabia. 19 Imams were purposively recruited from urban and rural settings and interviewed face-to-face. Interviews explored perceptions of religious leadership roles, opportunities for supporting diabetes self-care, and perceived challenges and barriers. Interviews were audio-recorded, and transcribed verbatim in Arabic. The analysis was performed using inductive thematic analysis to identify key themes related to Imams' perspectives on diabetes self-care promotion.

**Results:**

Five key themes emerged. First, Imams described their moral authority and community trust as a strong foundation for influencing health-related attitudes and behaviors. Second, participants identified multiple opportunities to support diabetes self-care through sermons, post-prayer talks, educational sessions, and individual counseling, particularly when health messages were framed within Islamic teachings. Third, substantial role ambiguity and lack of consensus existed regarding whether diabetes self-care promotion falls within Imams' professional responsibilities. Fourth, perceived barriers included limited health-related knowledge, concerns about credibility, anticipated congregant resistance, and skepticism about the effectiveness of behavior change efforts. Finally, participants highlighted the absence of institutional support, formal training, and structured collaboration with healthcare authorities as major constraints, despite general agreement that reinforcing adherence to medical advice aligns with their ethical role.

**Conclusions:**

Imams in Saudi Arabia perceive both meaningful opportunities and significant challenges in engaging as partners in diabetes self-care promotion. While high levels of trust and social influence position Imams as promising contributors to culturally grounded health initiatives, role ambiguity, training gaps, and lack of institutional support limit their involvement. Structured training, clear role delineation, and formal collaboration between health and religious institutions may enable effective, faith-aligned strategies to support diabetes self-care and improve health outcomes.

## 1 Introduction

Diabetes mellitus is a major and escalating public health challenge globally and within the Kingdom of Saudi Arabia (KSA). Worldwide, an estimated 589 million adults aged 20–79 lived with diabetes in 2024, with projections to rise to 853 million by 2050 due to demographic changes, urbanization, and lifestyle trends (1, 2). In Saudi Arabia, the prevalence of diabetes among adults is among the highest in the world, with recent national data reporting that approximately 23.1%−23.7% of adults are diagnosed with diabetes mellitus, equating to roughly 5.3 million adults, and projections indicating further increases if current trends persist (3). This substantial disease burden contributes to high morbidity and mortality and places considerable strain on healthcare systems through the management of chronic complications such as cardiovascular disease, nephropathy, and neuropathy (4).

Effective management of diabetes, including type 2 diabetes mellitus (T2DM), which constitutes the majority of cases depends heavily on self-care behaviors such as adherence to prescribed medications, dietary management, regular physical activity, blood glucose monitoring, and routine engagement with healthcare services. These self-care behaviors are critical determinants of glycemic control and reduced risk of long-term complications (5). Despite this, research in Saudi populations has identified suboptimal adherence to key self-management behaviors and varying levels of engagement in essential activities such as physical activity and blood glucose monitoring (5). Moreover, factors such as health and e-health literacy, cultural beliefs, socioeconomic status, and perceived social support significantly influence diabetes self-care engagement and outcomes (1).

Alongside these biomedical and psychosocial determinants, cultural and religious contexts play a profound role in shaping health behaviors in Saudi Arabia, where Islam is central to social life and moral identity. Religious beliefs and practices often inform daily routines, diets, and health-related decisions, elements that intersect directly with diabetes self-care practices (6). Previous research has highlighted that locus of health control beliefs, including a high belief in divine control of health outcomes, can influence medication adherence and self-management behaviors, indicating that religious beliefs may act as both facilitators and barriers to optimal diabetes care (7).

In this context, religious leaders, particularly Imams who deliver sermons and provide spiritual guidance in mosques occupy positions of influence and trust within communities. Historically, faith-based organizations and religious leaders have been recognized as important partners in health promotion efforts because of their broad reach, cultural legitimacy, and capacity to disseminate information that resonates with community values (8–10). Several studies conducted in Christian faith communities, particularly church based interventions in the United States, have demonstrated the effectiveness of faith centered health promotion programs in improving chronic disease prevention and self-management behaviors. These interventions addressed conditions such as diabetes, hypertension, obesity, and cardiovascular disease through health education sessions, lifestyle counseling, peer support, and community screening activities delivered in collaboration with pastors and church leaders. Evidence suggests that faith leaders can enhance trust, improve participation, and increase acceptance of health promotion messages among underserved populations (11–13).

Previous faith based interventions also reported barriers similar to those identified in the present study, including limited health literacy, insufficient medical training among religious leaders, concerns regarding boundaries between spiritual and medical guidance, and sustainability of programs. These challenges were commonly addressed through structured collaboration between healthcare professionals and faith leaders, provision of culturally tailored educational resources, training workshops, use of lay health educators, and integration of health promotion activities into existing religious gatherings and community structures (11, 14, 15). These findings suggest that faith institutions can serve as culturally trusted platforms for delivering health promotion interventions across diverse religious settings.

While much of the global evidence derives from church-based and multi-faith health promotion programs (e.g., cardiovascular risk reduction and hypertension interventions), which demonstrate that faith leaders and institutions can support health behavior change and mitigate health disparities, the specific role of mosque-based leadership in chronic disease self-management remains underexplored. A mosque-based, faith-integrated lifestyle intervention in rural Bangladesh, for instance, found that culturally tailored, mosque-led programs incorporating faith teachings with lifestyle guidance significantly reduced the incidence of T2DM among adults with prediabetes (16). Scoping reviews of mosque and faith-institution health promotion activities further underscore that culturally sensitive messaging and involvement of religious authorities can improve uptake of health behaviors and reduce communication barriers (17).

Despite these potential evidences, research specifically examining Imams' perspectives on diabetes self-care promotion in Saudi Arabia is limited. The study also identified cultural and religious misconceptions such as beliefs about divine destiny and reliance on God as barriers to self-care adherence.

This study aims to explore Imams' perspectives on their potential role as partners in promoting diabetes self-care in Saudi Arabia. It focuses on understanding their knowledge of diabetes, perceptions of self-care practices, and views on the opportunities and challenges associated with their involvement in supporting community members with diabetes. By examining these insights, the study seeks to inform culturally sensitive strategies for integrating religious leadership into diabetes self-care promotion, with the goal of enhancing adherence to self-management behaviors and improving health outcomes for people living with diabetes.

## 2 Methods

### 2.1 Study design

This study employed a qualitative descriptive design using semi-structured, in-depth interviews to explore Imams' perspectives on their potential role as partners in diabetes self-care promotion, as well as the perceived opportunities and challenges associated with this role. A qualitative approach was selected to capture participants' lived experiences, contextual insights, and nuanced interpretations of religious leadership within health-related domains. This manuscript represents one component of a broader qualitative project; however, the present analysis focuses specifically on role-related and institutional dimensions of diabetes self-care promotion.

### 2.2 Study setting and context

The study was conducted in the Kingdom of Saudi Arabia (KSA), where Imams serve as formal religious leaders within mosques and play a central role in guiding spiritual, moral, and social aspects of community life. Mosques represent key community spaces, and Imams often maintain close and sustained contact with congregants through religious services, sermons, and informal interactions. The cultural and religious significance of Imams positions them as potentially influential actors in community-based health promotion efforts, particularly for chronic conditions such as type 2 diabetes mellitus (T2DM), which is highly prevalent in KSA.

### 2.3 Participant recruitment and sampling

Participants were recruited using purposive sampling, targeting Imams who were actively involved in religious leadership roles within their communities. Eligibility criteria included: (1) currently serving as an Imam or delivering Friday sermons, (2) aged 18 years or older, and (3) willing to participate in an in-depth interview. Imams serving in both urban and rural settings were intentionally included to capture diversity in community contexts, religious practices, and experiences related to community engagement and health promotion.

Potential participants were identified and approached through professional, academic, and religious networks. Initial contact was made either via telephone or in person, depending on accessibility and participant preference. During recruitment, participants were provided with information regarding the study objectives, interview procedures, expected duration, voluntary nature of participation, and confidentiality measures before written informed consent was obtained.

Of the 27 Imams invited to participate, 19 agreed to take part in the study. Reasons for nonparticipation primarily included limited availability, competing professional responsibilities, or lack of interest in the topic. Recruitment and data collection continued concurrently until thematic saturation was achieved, defined as the stage at which no substantially new concepts, themes, or perspectives related to Imams' roles, opportunities, or challenges in diabetes self-care promotion emerged from subsequent interviews. Saturation was assessed through ongoing review and discussion of interview content and emerging themes among the research team.

### 2.4 Data collection

Data were collected through semi-structured, face-to-face interviews conducted by the principal investigator, who has formal training in qualitative research methods. An interview guide was developed based on the study objectives and relevant literature on faith-based health promotion and diabetes self-care (Supplementary Table S1). The guide included open-ended questions exploring:

- Perceived responsibilities of Imams beyond religious instruction
- Views on engaging with health-related topics, particularly diabetes
- Opportunities to support diabetes self-care within religious settings
- Perceived challenges, barriers, and limitations to such engagement
- Experiences advising congregants on health-related matters
- Views on collaboration with healthcare professionals and institutions

Three to four of the original Arabic transcripts and their English translations were co-reviewed by an additional neutral bilingual person to ensure accuracy. The interviewer wore culturally suitable clothes, such as the Saudi *Thoubs* and *Omamas*, to establish a respectful interaction. The investigator drew on an insider understanding of the cultural lifestyle of the participants and was particularly well-positioned to further understanding of the unique emotions expressed by body language and physical expressions that participants presented during the interviews.

### 2.5 Data management and translation

All interviews were audio recorded with participants' permission and transcribed verbatim in Arabic shortly after data collection. To ensure transcription accuracy and completeness, transcripts were reviewed against the original audio recordings by members of the research team. Field notes and contextual observations documented during interviews were also reviewed alongside transcripts to support interpretation of participants' responses and preserve contextual meaning.

For reporting purposes, selected participant quotations were translated from Arabic into English using a forward–backward translation process. Initial translations were conducted by bilingual researchers familiar with both the cultural and linguistic context of the study. Independent back translation was then performed to verify consistency, semantic equivalence, and preservation of intended meanings. Any discrepancies or ambiguities identified during translation were discussed among the research team and resolved through consensus to ensure conceptual accuracy and cultural appropriateness.

All interview data were anonymized prior to analysis. Identifying information, including names and mosque affiliations, was removed from transcripts to protect participant confidentiality and privacy. Each participant was assigned a unique identification code that was used throughout the analysis and reporting process. Audio files, transcripts, and related study materials were stored securely and accessed only by authorized members of the research team.

### 2.6 Data analysis

Data were analyzed using thematic analysis following the four-phase approach described by Vaismoradi et al. (18). Analysis was conducted iteratively alongside data collection. Transcripts were read repeatedly to achieve data immersion, and meaningful text segments were coded. Meaningful text segments relevant to the study objectives were identified and assigned initial codes. Codes were then systematically compared and grouped into categories, themes, and sub-themes based on conceptual similarities, recurring patterns, and relationships across participants' accounts.

The analytic process included initialization (familiarization and memo writing), construction (organizing codes into themes), rectification (reviewing and refining themes), and finalization (defining, interpreting, and reporting themes with supporting quotations). The interview guide was informed by the study aims and relevant literature, with selected questions adapted from Adedini et al. (19) and organized into general, cognitive, emotional, and social domains. Attention was given to preserving contextual meanings and participants' perspectives throughout the analytic process.

Two researchers independently participated in the coding and thematic development process to enhance the credibility and trustworthiness of the analysis. Initial coding was conducted separately, after which the researchers compared codes, themes, and interpretations through regular discussions. Any discrepancies or disagreements regarding coding or theme categorization were resolved through consensus and iterative review of the transcripts until agreement was achieved. This collaborative analytic process helped strengthen consistency, reduce subjective bias, and ensure accurate representation of participants' perspectives and experiences.

## 3 Results

A total of 27 Imams were approached for participation, of whom 19 agreed to take part in the study. Reasons for declining participation were primarily related to time constraints or doubts about the relevance of their perspectives. In line with Islamic regulations in Saudi Arabia, all participants were male, as the role of Imam is restricted to men. Participants' ages ranged from 26 to 55 years, with an average age of 43.1 years (SD = 8.3). All Imams were Saudi nationals, consistent with the requirements of the Ministry of Islamic Affairs (Table 1).

**Table 1:** Sociodemographic characteristics of participants (*N* = 19).

Characteristic	Category	*n* (%)
Educational background	Islamic studies	17 (89.5)
	Other (e.g., computer science, linguistics)	2 (10.5)
Age group	24–30 years	1 (5.3)
	31–40 years	7 (36.8)
	41–50 years	7 (36.8)
	51–60 years	4 (21.1)
Employment status	Full-time Imam	2 (10.5)
	Part-time Imam	17 (89.5)
Setting	Urban	15 (79.0)
	Rural	4 (21.0)

The majority of participants had received formal education in Islamic studies (89.5%). Several also reported holding additional professional positions, most commonly as school teachers or university lecturers, alongside their religious responsibilities. Five participants held postgraduate qualifications at the master's or doctoral level. Most Imams were affiliated with mosques in urban areas (79%), whereas four served exclusively in rural contexts, including villages and small towns. Two participants indicated that they were not officially assigned to a specific mosque at the time of data collection.

Regarding personal exposure to diabetes, most Imams reported having close family members, such as parents or siblings, diagnosed with diabetes, and one participant disclosed having type 2 diabetes mellitus himself. Reported estimates of Friday prayer attendance varied substantially, ranging from approximately 150–2,500 worshippers; however, attendance figures were not available for all mosques. It should be noted that official statistics on mosque attendance are not routinely collected.

Theme 1: Moral authority and community trust as a foundation for health promotion

Imams consistently described their position as one of trust, moral authority, and close social proximity to community members. Participants emphasized that congregants view Imams as respected leaders whose guidance extends beyond religious rituals to broader aspects of daily life.

“*Since many of them consider me a leader, this encourages me to set a good example and to respect the principles of sharia. I must act morally*.”

This trust was perceived as granting Imams privileged access to individuals at multiple levels public, interpersonal, and private, allowing them to influence attitudes and behaviors in ways that other professionals may not.

“*I can reach people through the Friday sermon, daily preaching after prayer, and personal communication*.”

Participants viewed this trust as a potential asset for promoting positive health behaviors, including diabetes self-care, particularly when messages are framed within Islamic values such as self-preservation and responsibility toward one's body.

Theme 2: Opportunities for supporting diabetes self-care within religious settings

Many Imams identified multiple avenues through which they could support diabetes self-care promotion. These opportunities included Friday sermons, post-prayer preaching sessions, educational talks, and individual counseling during informal interactions.

One participant articulated a multi-level framework for engagement:

“*At the public level through Friday sermons… at a more specific level through daily preaching… and at the personal level when the Imam speaks individually to people*.”

Participants noted that religious teachings already emphasize moderation, self-care, and avoiding harm, which could naturally be linked to diabetes self-care behaviors.

“*By emphasizing that taking proper care of one's health is the right of the body, I can link Islamic phrases with doctors' instructions*.”

Several Imams expressed willingness to collaborate with healthcare providers and saw themselves as potential bridges between medical advice and community acceptance.

Theme 3: Lack of consensus and role ambiguity among Imams

Despite recognizing their influence, Imams expressed divergent views regarding whether diabetes self-care promotion falls within their professional responsibilities. Some viewed health promotion as compatible with religious leadership, while others believed it lay outside their mandate.

“*I don't think it is appropriate to mix health matters with religious activities*.”

Those holding restrictive views emphasized that mosques are primarily spaces for spiritual guidance, not health education, and expressed concern about diluting religious content.

“*The Friday sermon should guide people to their creator and the afterlife. That is the primary role of the Imams*.”

In contrast, others argued that the prevalence of diabetes warrants attention within religious discourse.

“*Today diabetes is very common… I think it must be in our consideration in Friday sermons*.”

This lack of consensus created uncertainty and inconsistency in health-related engagement across religious settings.

Theme 4: Perceived barriers to engaging in diabetes self-care promotion

Imams identified multiple barriers that limited their ability or willingness to engage in diabetes self-care promotion. A major concern was lack of professional qualification, with many expressing fear of providing incorrect information.

“*Your role as an Imam does not give you a position to give advice about health issues*.”

Some participants worried about negative reactions from congregants, including resistance to health messages or skepticism toward medical information delivered by religious leaders.

“*People primarily come to the mosque for religious purposes and may not be receptive to health messages*.”

Another prominent barrier was perceived community resistance to behavior change. Several Imams expressed pessimism regarding congregants' willingness to adopt healthier lifestyles, regardless of who delivers the message.

“*Most people don't like restrictions… even if we dedicate the sermon to health care, it will not make a difference*.”

These perceptions contributed to disengagement and reluctance to invest effort in health promotion.

Theme 5: Absence of institutional support and training

While Theme 3 reflects differing opinions among Imams regarding whether health promotion falls within their professional and religious responsibilities, Theme 5 specifically captures the structural and institutional conditions required to support such involvement. Participants distinguished personal willingness and role perception from the practical need for formal training, organizational guidance, and collaboration with healthcare institutions to ensure safe and effective engagement in diabetes self-care promotion. Participants unanimously highlighted the lack of formal training, guidance, or institutional support to assist Imams in health promotion roles. Most reported that they had never received structured education related to health or chronic disease management.

“*We need courses and workshops… cooperation between the Ministry of Health and the Ministry of Islamic Affairs would be helpful*.”

Some expressed concern that additional training would impose further burdens, as many Imams already balance religious duties with other employment.

Others emphasized that prevailing stereotypes viewing Imams solely as religious figures undermine their credibility in health-related matters.

“*People won't accept medical information that's provided by Imams who aren't doctors*.”

Despite these challenges, most Imams agreed that directing congregants to healthcare services and reinforcing adherence to medical advice aligns with their ethical responsibilities.

## 4 Discussion

This study examined *Imams'* perspectives on their potential role as partners in diabetes self-care promotion in Saudi Arabia, revealing key opportunities and barriers rooted in religious authority, community trust, role ambiguity, and institutional support. The findings advance understanding of how faith leadership might be harnessed for chronic disease self-management in Muslim contexts and align with broader evidence on faith leaders' influence in health promotion.

A central finding was that Imams perceive themselves as trusted moral authorities with unusually high social proximity to congregants. Participants described their influence as extending beyond ritual worship into everyday life, enabling them to shape attitudes and behaviors at public, interpersonal, and individual levels. This resonates with study showing that faith leaders often hold strong influence on health behaviors within their communities due to their moral status and interpersonal reach. Studies across faith contexts have emphasized that religious leaders can shape health norms by serving as role models and trusted messengers, thereby facilitating awareness and behavior change for chronic disease risk factors and health behaviors (20).

Linking health promotion to religious values as participants suggested by framing diabetes self-care within Islamic teachings on self-preservation and responsibility also aligns with evidence that culturally and spiritually congruent health messages are more readily accepted by faith communities. Faith-integrated health interventions, including mosque-based lifestyle programs, have shown beneficial outcomes for diabetes prevention when health guidance is embedded within religious frameworks (16).

The Imams' identification of multiple avenues for outreach including Friday sermons, daily preaching, educational talks, and informal one-on-one interactions reflects the broad potential leverage points for health messaging in mosque environments. This is consistent with literature documenting the diverse platforms faith leaders can use to disseminate health information and foster healthy norms. Religious leaders often engage at multiple socio-ecological levels, shaping social and environmental contexts in addition to individual behavior (21).

Moreover, linking diabetes self-care principles to existing religious teachings on moderation and avoidance of harm may enhance the cultural relevance of health promotion. Evidence suggests that health messages attached in familiar theological or scriptural constructs can be more convincing and reduce resistance to behavioral guidance (22).

A clear barrier identified was role ambiguity, with Imams expressing divergent views on whether diabetes self-care promotion falls within their professional mandate. Some regarded health promotion as consonant with religious leadership, while others viewed it as outside the scope of mosque functions. This reflects broader challenges documented in faith-health collaborations, where differing perceptions of appropriate roles for religious figures can hinder consistent engagement. A study showed that faith leaders resist health promotion if they perceive it as reducing spiritual content or overstepping traditional boundaries (23). Without clear role definitions and shared expectations, mosque-based health messaging may remain inconsistent across communities, undermining efforts to systematically address chronic diseases like diabetes.

Consistent with prior study, the Imams' self-reported lack of professional health knowledge emerged as a substantial barrier. Many expressed concern about providing inaccurate or non-evidence-based advice, which may reduce credibility if combined with medical misinformation. This mirrors findings in other contexts where religious leaders emphasize the need for proper training and collaboration to avoid disseminating erroneous information, particularly when addressing complex health issues (24). Faith leaders in other studies have similarly noted that beliefs and sociocultural norms can limit acceptance of health guidance even when delivered by trusted figure (23).

The lack of structured training, guidance, and institutional support was a common theme among Imams engaged in health promotion roles. Participants emphasized that mosques and religious institutions currently lack formal mechanisms for health education and expressed the need for partnerships with healthcare authorities to develop appropriate curricula and training workshops. This need aligns with the broader literature advocating for capacity-building initiatives that equip faith leaders with accurate health information and communication skills (25). Training not only enhances knowledge but also legitimizes the role of religious leaders in health promotion by bridging gaps between spiritual authority and medical expertise. Effective collaborations between mosques and health agencies have been recommended in other health fields to strengthen community outreach and reduce disparities (25).

Our findings are consistent with experiences from church based and other faith centered health promotion interventions conducted in diverse settings. Previous programs targeting diabetes, hypertension, obesity, and cardiovascular disease have demonstrated that faith leaders can successfully support health behavior change when provided with structured training, culturally tailored educational resources, and collaboration with healthcare professionals. Similar barriers including role ambiguity, limited medical knowledge, and concerns regarding credibility were addressed through interdisciplinary partnerships, integration of health activities into existing religious gatherings, and involvement of healthcare workers in developing and delivering educational content (11–15). These experiences suggest that sustainable mosque based diabetes self-care initiatives in Saudi Arabia may benefit from formal collaboration between religious institutions and public health authorities to ensure both cultural relevance and medical accuracy.

The findings of this study also have important implications for health policy and community-based diabetes prevention strategies in Saudi Arabia. By identifying practical entry points such as Friday sermons, individual counseling, educational sessions, and collaboration with healthcare institutions, the study highlights how Imams may contribute to culturally aligned diabetes self-care promotion within communities. Similar to community health workers and local community leaders in other public health programs, trained religious leaders may serve as trusted intermediaries who reinforce health messages, encourage healthcare seeking behaviors, and improve community engagement in chronic disease prevention and management. Integrating religious leaders into broader noncommunicable disease strategies through structured training programs and institutional collaboration may therefore strengthen primary healthcare outreach and support culturally sensitive health promotion efforts.

### 4.1 Strengths and limitations

This study has several important strengths. First, it provides novel, in-depth qualitative insight into Imams' perspectives on diabetes self-care promotion in Saudi Arabia, a topic that remains underexplored in the literature. By focusing directly on religious leaders who hold significant moral authority and social influence the study addresses a critical gap in understanding how faith-based structures may contribute to chronic disease self-management in Muslim contexts. Second, the study included Imams from diverse age groups, educational backgrounds, and mosque settings, including both urban and rural areas. Third, the use of semi-structured interviews enabled participants to articulate their views freely, allowing for nuanced exploration of opportunities, challenges, and ethical concerns related to health engagement. Finally, the study's focus on institutional and structural factors, such as training needs and inter-ministerial collaboration, extends beyond individual attitudes and provides actionable insights for policy and practice.

Several limitations should be acknowledged. First, the study included a relatively small sample size (*N* = 19), which is typical for qualitative research but limits generalizability. Second, the sample was predominantly urban, reflecting the distribution of participating mosques. Imams serving exclusively in rural areas may face different community dynamics, resource constraints, and health beliefs, which could influence their engagement in diabetes self-care promotion. Third, participation was voluntary, raising the possibility of self-selection bias. Fourth, as with all interview-based studies, the findings rely on self-reported perceptions, which may be influenced by social desirability bias. Finally, the study captured only the perspectives of Imams and did not include the views of congregants, healthcare professionals, or policymakers. Triangulating these perspectives in future research would provide a more comprehensive understanding of the feasibility and acceptability of involving religious leaders in diabetes self-care promotion.

## 5 Conclusion

This study highlights the significant potential of Imams to serve as partners in diabetes self-care promotion in Saudi Arabia, grounded in their moral authority and community trust. While many Imams recognized opportunities to support diabetes self-management through religious settings, substantial challenges were also identified, including role ambiguity, limited health-related training, perceived community resistance, and the absence of institutional support. Addressing these barriers through structured training, clear role, and formal collaboration between health and religious authorities may enable culturally appropriate, faith-aligned strategies that strengthen diabetes self-care and improve health outcomes among people living with diabetes.

## Data Availability

The original contributions presented in the study are included in the article/Supplementary material, further inquiries can be directed to the corresponding author.
