# Supplementary material for: Religious leaders as partners in diabetes self-care promotion: opportunities and challenges from Imams' perspectives in Saudi Arabia
Source: Front Public Health. 2026 Jun 11;14:1830915. doi: 10.3389/fpubh.2026.1830915 (PMC13294470; doi:10.3389/fpubh.2026.1830915)
Supplement: Supplementary file 1 [file Table_1.docx]

**Religious Leaders as Partners in Diabetes Self-Care Promotion: Opportunities and Challenges from Imams’ Perspectives in Saudi Arabia**

Bandar S. Alharbi^1*^, Majed M. Aljabri^1^

^1^Community and Psychiatric Mental Health Department, College of Nursing, King Saud University, Riyadh 12375, Saudi Arabia

Table S1: *Guided Interview Questions*

| Dimensions | Questions | | Probes |  |
| --- | --- | --- | --- | --- |
| General | For how long have you been assigned as an Imam? | |  |  |
|  | How many congregations pray in your Mosque? | |  |  |
|  | Can you tell me more about the congregation? Are any of them not Arabic? If so, how do you deal with them? | |  |  |
| Cognitive | Tell me what you know about diabetes and self-management | |  |  |
|  | Who do you think is at greatest risk of developing diabetes-related complications? Why? | |  |  |
|  | For how long you think (exercise, diet…etc.) can improve diabetes outcomes? | |  |  |
|  | In your opinion, what are the causes of T2DM? | |  |  |
|  |  | |  |  |
| Emotional |  | | Can you provide examples of what you do? |  |
|  | Can you share with me how Islamic beliefs might play a positive or negative role for people with diabetes? | |  |  |
|  | How do you feel about diabetes self-care as a lifetime procedure for those with T2DM? | |  |  |
|  | What do you think are the reasons that have prevented people with T2DM from adhering to diabetes self-care? | |  |  |
|  | What are some things that would motivate the congregants to adhere to diabetes self-care? | |  |  |
| Social |  |  |  |  |
|  | In your opinion, what are the benefits of being healthy? | | Can you tell me how that will contribute to society? | |
|  | What advice would you give to patients diagnosed early with T2DM? Why? | |  | |
|  | What part of diabetes self-management do you think you focus on when providing advice? Why? | |  | |
|  | What do you think is your role in helping patients with T2DM manage their condition? | |  | |
|  |  | |  | |
